# Cholangioscopy and double guidewires facilitate a difficult endoscopic ultrasound-guided gastroenterostomy

**DOI:** 10.1055/a-2743-2487

**Published:** 2026-01-08

**Authors:** Jiahuan Liu, Shuai Bai, Jia Xie, Jinlin Yang, Rui Wang

**Affiliations:** 1 34753 Department of Gastroenterology and Hepatology, West China Hospital, Sichuan University, Chengdu, China

Endoscopic ultrasound-guided gastroenterostomy (EUS-GE) with electrocautery-enhanced lumen-apposing metal stents (LAMSs) is a promising minimally invasive approach for benign or malignant gastric outlet obstruction (GOO). Guidewire-assisted oroenteric catheters (OECs) or balloon catheters are commonly used to distend the small bowel for puncture. However, failed guidewire passage may complicate the procedure.

**Fig. 1:**
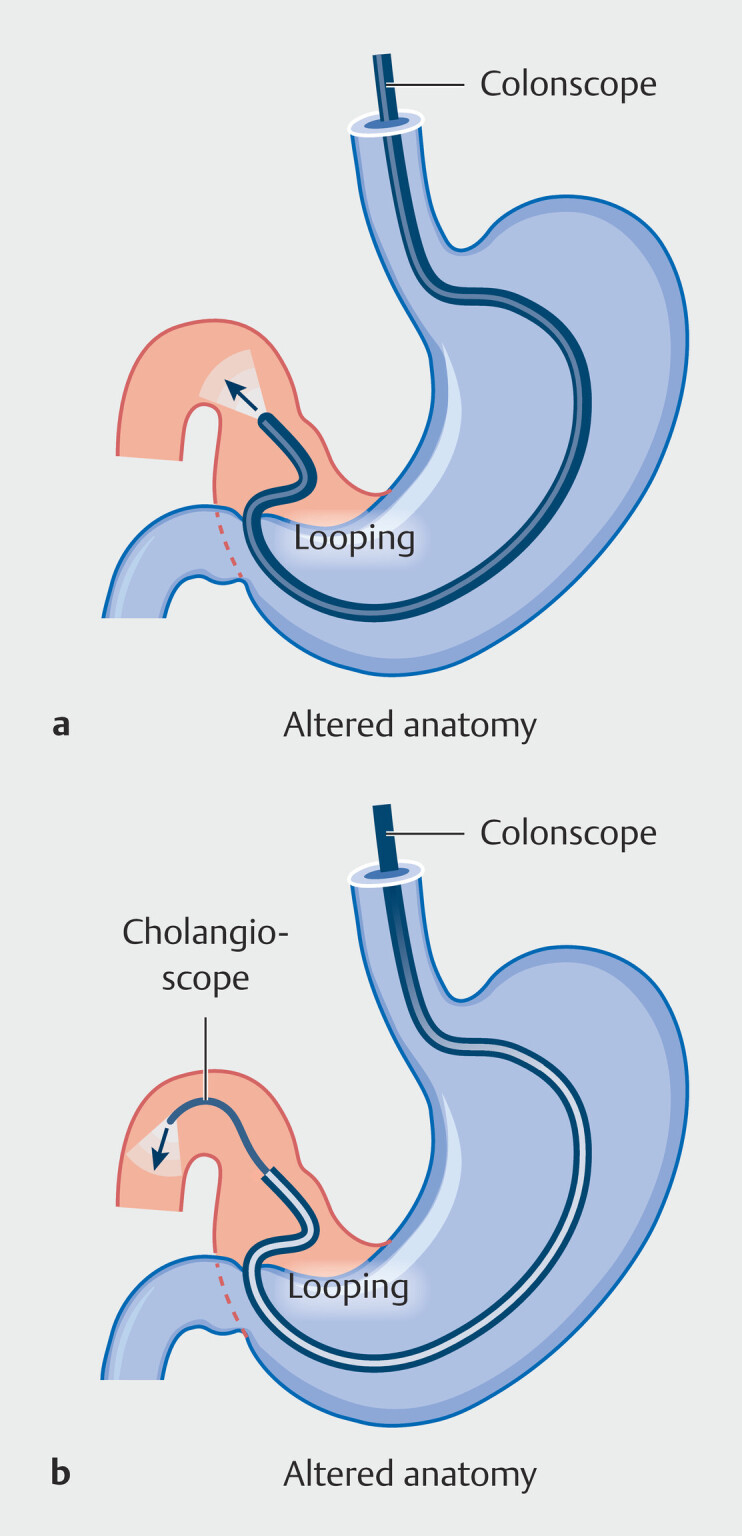
**a** Schematic illustration showing severe scope looping in a patient with gastric outlet obstruction and surgically altered anatomy after liver transplantation and hilar bile duct plasty, preventing endoscopic passage to the pylorus despite abdominal compression and repositioning. **b** Schematic illustration of a cholangioscope advanced through the working channel of a colonoscope to traverse the pylorus and enter the duodenal bulb, overcoming the limitation of severe scope looping in surgically altered anatomy.

**Fig. 2:**
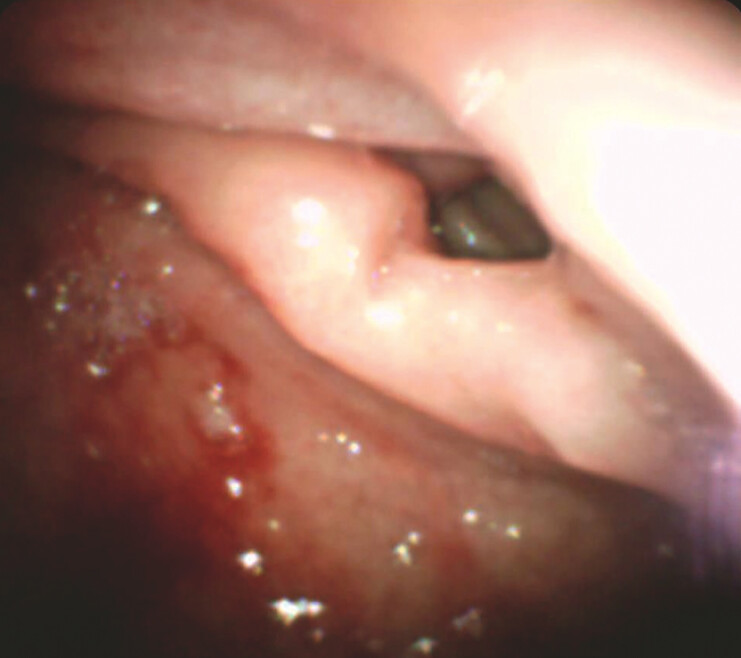
The hidden pathway to the jejunum located at the 1 o’clock position in the duodenal bulb, obscured by altered gastric anatomy.

**Fig. 3:**
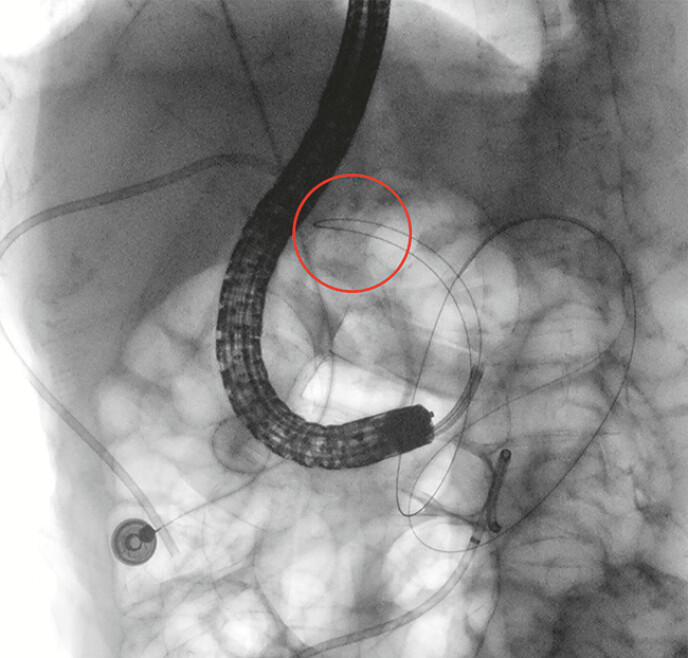
An angled-tip hydrophilic guidewire was inserted, navigating a sharply curved duodenal bulb with the maximal bend highlighted by a red circle, and advancing distally into the jejunum.

**Fig. 4:**
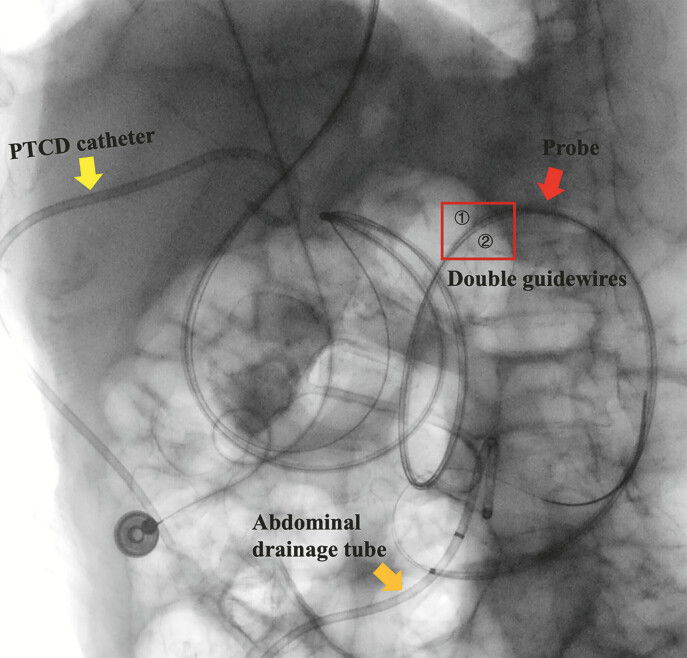
A fluoroscopic image showing dual-guidewire access established using an 8.5 Fr bougie catheter.

**Fig. 5:**
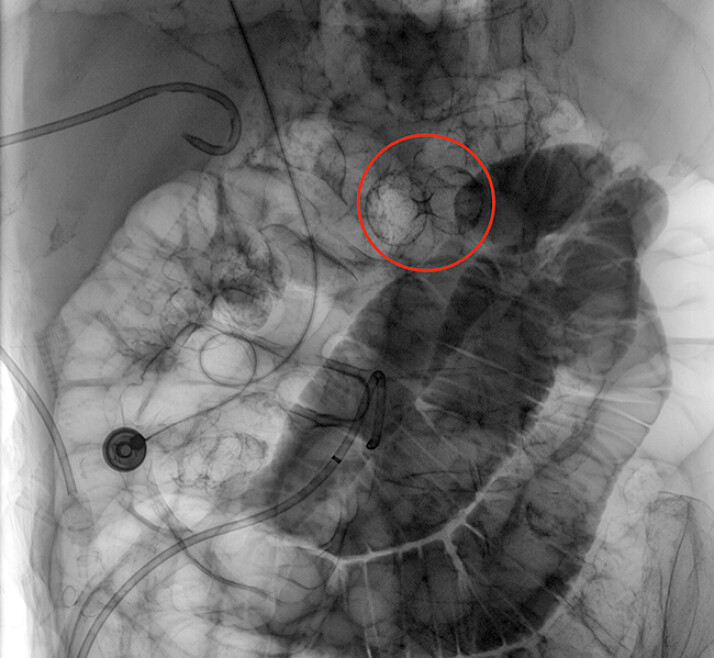
Successful deployment of a LAMS between the stomach and the jejunum under combined guidance of dual-guidewire access and cholangioscopy, with the LAMS position highlighted by a red circle. LAMS, lumen-apposing metal stent.

A 59-year-old woman with GOO after liver transplantation and hilar bile duct plasty was scheduled for EUS-GE. However, multiple endoscopes (ultra-slim endoscope, enteroscope, gastroscope, and colonoscope) failed to reach the pylorus due to severe scope looping, despite abdominal compression and repositioning ( Fig. 1 **a** ). To overcome this situation, a 9 Fr cholangioscope (IMAX, Nanwei Medical) was advanced through the 3.7 mm channel of a colonoscope, traversed the pylorus, and entered the duodenal bulb ( Fig. 1 **b** ). The pathway was visualized hidden at a 1 o’clock position in the duodenal bulb ( Fig. 2 ). Under direct vision, an angled-tip hydrophilic guidewire (RF*PA35263M, Terumo) was advanced, followed by the cholangioscope into the jejunum ( Fig. 3 ). Subsequently, a Boston Scientific Jagwire high-performance guidewire (M00556580) was inserted via an 8.5 Fr bougie catheter to secure dual-wire access ( Fig. 4 ). Then, a 7 Fr OEC was used to infuse dye-mixed saline. Finally, a 15-mm Hot AXIOS LAMS (Boston Scientific) was successfully deployed under a linear echoendoscope guidance, with positioning confirmed by endoscopy, EUS, and fluoroscopy ( Fig. 5 , Video 1 ). The second guidewire was then withdrawn. The patient resumed a liquid diet 3 days later.

Cholangioscopy-assisted EUS-GE in a patient with surgically altered anatomy. The video highlights overcoming severe scope looping by cholangioscopy through a colonoscope, precise navigation using angled-tip guidewires under direct vision, establishment of dual-guidewire access for the procedural backup, and safe deployment of a LAMS with real-time confirmation, minimizing fluoroscopy use.Video 1

This case highlights key technical advantages that enabled successful EUS-GE in surgically altered anatomy. First, cholangioscopy within a colonoscope increased rigidity and reach, overcoming severe gastric looping. Second, angled-tip guidewires enabled precise navigation under direct vision by cholangioscopy. Third, real-time visualization allowed the confident identification of the jejunal lumen without contrast or radiation. Finally, dual-guidewire access offered a backup for feeding tube placement and enabled repeat cholangioscopy to confirm LAMS deployment, reducing reliance on fluoroscopy.

Endoscopy_UCTN_Code_TTT_1AO_2AN

